# Do socio-cultural factors influence medical students’ health status and health-promoting behaviors? A cross-sectional multicenter study in Germany and Hungary

**DOI:** 10.1186/s12889-016-3228-1

**Published:** 2016-07-15

**Authors:** Henna Riemenschneider, Péter Balázs, Erika Balogh, Axel Bartels, Antje Bergmann, Károly Cseh, Nora Faubl, Zsuzsanna Füzesi, Ferenc Horváth, István Kiss, Jörg Schelling, András Terebessy, Karen Voigt

**Affiliations:** Department of General Practice, Medical Clinic 3, University Hospital Carl Gustav Carus, Technische Universität Dresden, Fetscherstr. 74, 01307 Dresden, Germany; Department of Public Health, Faculty of Medicine, Semmelweis University Budapest, Nagyvárad tér 4, H-1089 Budapest, Hungary; Department of Public Health Medicine, University of Pécs Medical School, Szigeti str 12, H-7624 Pécs, Hungary; Department of Behavioral Sciences, University of Pécs Medical School, Szigeti str 12, H-7624 Pécs, Hungary; Department of General and Family Medicine, Medical Faculty, Ludwig-Maximilians-Universität München, Pettenkoferstr. 8a, 80336 Munich, Germany

**Keywords:** Health behavior, Health status, Health promotion, Socio-cultural factors, Medical students, Multicenter study

## Abstract

**Background:**

Physical and mental health is important for coping with the high requirements of medical studies that are associated with a higher risk for severe stress, insomnia, smoking, harmful alcohol consumption and easier access to drugs. Health behaviors of medical students influence not just their own health but also the health of their future patients. We examined whether socio-cultural factors can explain differences in students’ health status and health-promoting behaviors.

**Methods:**

A multicenter cross-sectional survey in Germany (Dresden, Munich) and Hungary (Budapest, Pécs) enclosed international medical students in their 1st, 3rd and 5th academic years. The students were invited to voluntarily and anonymously complete a questionnaire on different aspects of health behavior during obligatory seminars and lectures in 2014. The response rate of the total sample was 56.2 % (n = 2935); the subgroup analysis enclosed data of German (n = 1289), Hungarian (n = 1057) and Norwegian (n = 148) students.

**Results:**

A high number of Norwegian students (84.5 %) assessed their health status as *very good/excellent*. In comparison, only 60.3 % of the Hungarian and 70.7 % of the German participants reported a *very good/excellent* health status. The distributions were comparable between the study sites. Although gender, financial situation and nationality were significant health status predictors, they could explain only 8.2 % of the total variance of health status in the multivariable model. A comparably high number of Hungarian students (95.3 % vs. 67.4 % German and 56.7 % Norwegian) reported that they can currently do *a lot/very much* for their health. In contrast, a significant number of Norwegians (73.0 % vs. 63.7 % Hungarian and 51.5 % German) reported that they currently do *a lot/very much* for their health (chi^2^-test, p ≤ 0.001). Financial situation, study site and study year were the strongest predictors for health promotion activities (Nagelkerkes R^2^ = 0.06).

**Conclusions:**

Based on our study, gender and study year played only a minor role in the health status and health promotion beliefs and activities of medical students. Structural (study site) and somewhat socio-cultural factors (nationality, financial situation) mainly explained the differences regarding health promoting behaviors. Obligatory, free-of-charge courses for health promotion (activity and relaxation) should be included in study curriculums.

## Background

The physical and mental health of medical students is important for coping with the high requirements of medical studies that are associated with a higher risk for stress, insomnia, smoking, harmful alcohol consumption and easier access to drugs that can lower the barriers to substance abuse [1–4]. Health status, attitudes and health behavior also influence the management of the requirements of later work life. Health behavior of physicians and their attitudes to risk management may also affect the interaction with patients (contents, frequency and success of counseling) and the sensitivity to detect risk factors [5–10].

During their studies, medical students gain a better health knowledge than other students or the general population. That knowledge does not, however, directly transfer to the students’ own preventive behaviors [11]. Based on European and U.S. studies, large proportions of medical students report a good health status and positive health behaviors [12–15]. Nevertheless, medical studies are also associated with mental health problems [16] and negative behaviors and practices [11–13, 17–19]; e.g. the consumption of legal and illegal substances that may even exceed that of the general population [17, 18].

Previous studies mainly detect differences in health behavior among medical students with regard to gender, age or academic year. However, multidimensional factors influence health, and some studies indicate possible differences in health behavior and self-assessed quality of life depending on country of origin [14, 15]. Further socio-cultural factors, e.g. ethnicity, religion and wealth [20, 21], are seldom considered as influencing factors for health status or health (−promoting) behaviors in studies among medical students. Considering the increasing mobility of students due to travel and exchange programs during their studies and also labor based (physician) migration [22], these factors become increasingly valuable in the field of health promotion.

Reflecting biopsychosocial models for explanation of health, a) personal (i.e. genetic disposition, physical-mental condition and ethnicity), b) structural (i.e. socioeconomic structures, educational offers, health care system, social support) as well as c) behavioral factors (i.e. health habits, health beliefs, self-efficacy expectancies, coping competences) affect health behaviors and health status. According to social-cognitive approaches, health behavior is influenced by cognitive, emotional and motivational factors that depend on multiple social or socio-demographic factors, i.e. age, gender, social status and social networks [23, 24]. Looking at sociocultural factors can help to explain differences in health status and health-promoting behaviors.

### Aim of the study

The aim of this cross-sectional multicenter study is to bring new information concerning health status and health behaviors in an international sample of medical students affiliated to different cultural, social and economic backgrounds, during the training years either abroad or in their home countries. We examined to what extent medical students assess being able to do a lot for their health, and how they actually promote their health. We also investigated the associations of health status and health promoting behavior in correlation with academic years, gender, age, and with other socio-cultural factors such as country of origin, living situation, financial situation and religiousness. Novel information regarding adjustable (and non-adjustable) socio-cultural and setting factors associated with students´ health and health behavior can be applied for developing recommendations for health promotion activities and interventions for (medical) students.

## Methods

### Study design and survey instrument

This project was designed as a cross-sectional multicenter study in collaboration with the departments of General Practice at Technische Universität Dresden and Ludwig-Maximilians-Universität Munich (Germany), Public Health at Semmelweis University Budapest and departments of Public Health and Behavioral Sciences at University of Pécs (Hungary).

The 9-page-survey questionnaire for medical students was developed in a multiple Delphi process carried out by all collaborative partners. The questionnaire was largely based on validated instruments (e.g. SF-36 [25] for measuring health status and health promotion) and previous surveys of Technische Universität Dresden and Semmelweis University [14, 17, 19]. Regarding the international target group, the questions were adjusted to the specific regional and cultural conditions.

The questionnaire was first developed and approved in English, and then translated to German and Hungarian, including parts of the used validated instruments in the original wording for all three languages. To control the feasibility of the questionnaires in all three languages, pretests were conducted on the campuses in February 2014 in German, Hungarian, and in English amongst international study groups (n = 131). Based on the first pretest phase, minor revisions were done to optimize the questionnaire in all language versions. After the second pretest phase the questionnaires were finalized. The survey protocol ensured that the procedure was the same in all four study sites (Dresden, Munich, Budapest, Pécs).

The final questionnaire included questions on socio-demographics, on various aspects of health behavior (e.g. health status, sleep, physical activity, medication, vaccination, diet, quality of life), and on risk behavior (e.g. consumption of legal and illegal substances, sexual behavior).

### Study participants and setting

Medical students in their 1st, 3rd and 5th academic years were invited to participate voluntarily and anonymously in the study during mandatory seminars/tutorials and lectures, targeting a full sample survey in order to keep the selection bias as low as possible. The study purpose, anonymity and voluntariness and the consent of participation by filling in the questionnaire were declared in the survey cover letter. According to the ethics approval, the data were recorded anonymously; conclusion on individual persons is not possible. The questionnaires were distributed to all students to ensure anonymity of non-participants. After filling out the questionnaires (duration about 20 min), the questionnaires were collected in boxes at the doors. The data collection was conducted in all four study centers (universities in Dresden, Munich, Budapest and Pécs) in 2014, targeting ca. 5000 registered students.

In addition to German and Hungarian students, a large group of international students were included in the survey: ca. 40 % of students at medical faculties in Hungary originate from different countries, explained by existing study programs for medicine in Hungarian, German and English language. German, Hungarian and Norwegian students were the largest subpopulations in our sample.

### Statistical analysis

The data analysis was performed using SPSS 22.0. Pearson’s chi^2^-tests were used to determine whether there were significant differences between frequencies regarding different subgroups. Bonferroni’s adjusted t-test for unpaired samples was used for comparing means of metric data (e.g. age) of the different subgroups. For examining correlations between ordinal data, the Spearman correlation test was applied. Logistic regressions were executed to meet the complexity of influencing factors (based on bivariate analysis) on health status and health promotion as well as to reduce intercorrelating effects. In order to compare the influence of nationalities in the regression models, Norwegian students were selected as a reference group since they showed the highest proportions of answer option “excellent”. The influence of the study site (abroad vs. home country) on health status and health promotion was only examined among German students because they were the only larger subsample found at all study sites.

## Results

### Description of the sample

A total of 2935 students of 65 different nationalities participated in our multicenter study (response rate: 56.2 %). Because of the sample size, only students from the three largest student groups: Germans (*N* = 1289), Hungarians (*N* = 1057) and Norwegians (*N* = 148), were included in the subgroup analysis of this study.

There were significant differences regarding age, academic year, living and financial situation, and religiousness among the subpopulations (Table 1). The distribution of genders was comparable in all subpopulations. The mean age of Norwegian students was significantly higher, and accordingly the number of Norwegians in their later academic years was higher. More often than the Germans and Hungarians, Norwegian students also reported living alone, having fewer financial problems and being less religious.

**Table 1:** Socio-cultural characteristics of the sample

Parameters	Statistics	Total sample (*N* = 2935)	German students (*N* = 1289)	Hungarian students (*N* = 1057)	Norwegian students (*N* = 144)	Test on differences
Age	Mean ± SD	22.5 ± 3.3	22.9 ± 3.6	21.6 ± 3.6	23.9 ± 2.7	Multiple two sample t-test (Bonferroni adjusted): *p* ≤ 0.001
Gender: female	N (%)	1797 (61.2)	784 (61.0)	674 (63.9)	94 (63.9)	Pearson's chi^2^ test: *p* ≥ 0.05
Academic year:	N (%)					Pearson's chi^2^-test: *p* ≤ 0.001
First		1260 (42.9)	626 (48.3)	437 (41.3)	31 (20.9)	
Third		891 (30.4)	371 (28.6)	306 (28.9)	65 (43.9)	
Fifth		667 (22.7)	264 (20.4)	270 (25.5)	42 (28.4)	
Living situation: alone	N (%)	896 (30.5)	462 (35.8)	135 (12.8)	78 (53.1)	Pearson's chi^2^-test: *p* ≤ 0.001
Financial situation:	N (%)					Pearson's chi^2^-test: *p* ≤ 0.01
No/hardly any problems		2070 (70.5)	937 (73.6)	716 (69.2)	122 (84.1)	
Sometimes problems		580 (19.8)	716 (19.5)	225 (21.8)	16 (11.0)	
Often/daily problems		219 (7.5)	88 (6.9)	93 (9.0)	7 (4.8)	
Religiousness:	N (%)					Pearson's chi^2^-test: *p* ≤ 0.001
Not at all/not very religious		1543 (52.6)	760 (59.7)	442 (43.9)	114 (77.6)	
Moderate religious		967 (32.9)	399 (31.3)	415 (41.3)	26 (17.7)	
Very religious		328 (11.2)	114 (9.0)	149 (14.8)	7 (4.8)	

### Health status and associated factors

**Fig. 1:**
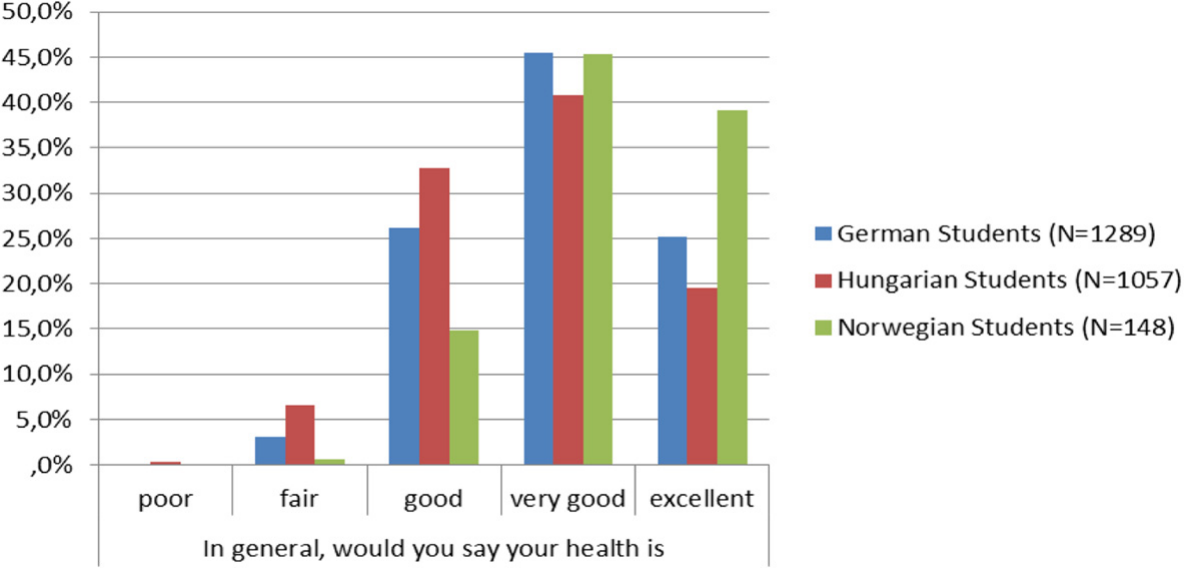
Self-reported health status of students with different nationalities

The majority of all participating medical students described their health as *good* (28.3 %), *very good* (43.5 %) or *excellent* (23.6 %). Significant differences (chi^2^-test, *p* ≤ 0.001) were detected depending on the nationality: among Norwegians the largest (84.5 %) and among Hungarians the lowest (60.3 %) proportions of students assessed their health status as *very good/excellent* (Fig. 1).

#### Socio-demographic factors

We detected no significant correlations between assessment of health status and age (r_spearman_ = −0.013/*p* ≥ 0.05). Significant differences were observed depending on gender (chi^2^-test, *p* ≤ 0.001): Whereas 30.2 % of male students reported an *excellent* health status, only 21.2 % reported a *good* health status. In comparison, fewer female students (19.6 %) assessed their health status as *excellent* but a higher number of females (32.7 %) considered it to be *good*.

#### Study context

The distributions regarding the assessment of health status as *very good/excellent* were similar at all study sites: between 64.6 % (Budapest) and 71.8 % (Munich). (These differences were small but significant based on the large sample size). We detected no significant correlations between assessment of health status and study year (r_spearman_ = −0.007*p* ≥ 0.05).

The analysis of the health status of the students with regard to their nationalities and choice of study site revealed descriptive differences only among the German student subgroup (chi^2^-test, *p* ≤ 0.05): A significantly higher number of Germans studying at a Hungarian site (73.0 % in Pécs and 74.8 % in Budapest) described a *very good/excellent* health status than German students studying in Germany (68.0 % in Dresden and 71.8 % in Munich). There were no significant differences of health status observed in Norwegian or Hungarian students in dependency of their places of study (Pécs, Budapest).

#### Socio-cultural factors

**Fig. 2:**
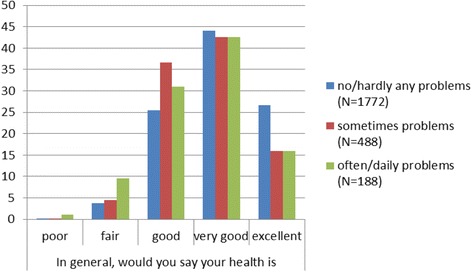
Self-reported health status and financial situation of medical students

There were also differences in health status depending on the living situation: significantly more students living alone reported *excellent* health (29.4 %) compared to students living together with at least one person (21.5 %). Slightly fewer students describing themselves as very religious (compared to moderate religious or not very/not religious) assessed their health as *excellent/very good* (62.4 % vs. 67.1 % vs. 69.1 %), but these differences appeared not significant (chi^2^-test, *p* ≥ 0.05). A weak but significant correlation was found between financial situation and health status (r_spearman_ = −0.138/*p* ≤ 0.001). Medical students who are in problematic financial situations on a frequent/daily basis reported a *poor/fair* health (chi^2^-test, *p* ≤ 0.001) significantly more often. Higher proportions of students with no or hardly any financial problems assessed their health as *very good/ excellent* (Fig. 2).

#### Predictors of health status in a multivariable model

All the significantly associated variables of the bivariate analysis (see above) were included in a multinominal logistic regression model. Different predictors were detected depending on answer options (*poor/fair* to *excellent* health). The chance of *good* to *excellent* health compared to *poor/fair* health was significantly increased in students who reported *no/hardly any* (OR ≥ 2.4) or *sometimes* (OR ≥ 2.5) financial problems (Table 2). Hungarian nationality was significantly associated with a decreased chance of *very good* (OR = 0.081) or *excellent* (OR = 0.052) health status. Male gender reduced the chance of *good* compared to *poor* health (OR = 0.637) but did explain differences between *very good/excellent* compared to *poor* health status. However, these significant predictors could explain only 8.2 % (Nagelkerkes R^2^ = 0.082) of the total variance of health status in the model.

**Table 2:** Predictors of health status based on a multinominal regression model

In general, would you say your health is…^a^		Significance	Exp(B)	95 % confidence interval Exp(B)	
				Lower limit	Upper limit
Good	Constant	,030			
	Gender: male	,040	,637	,414	,980
	Nationality (ref. Norwegian)				
	German	,585	,548	,063	4,739
	Hungarian	,143	,218	,028	1,677
	Financial situation (ref. often/daily problems)				
	No/hardly any financial problems	,003	2,379	1,334	4,242
	Sometimes problems	,002	2,966	1,494	5,886
	Study place (ref. Budapest)				
	Dresden	,683	,818	,313	2,140
	Munich	,843	,900	,318	2,548
	Pécs	,193	1,403	,843	2,337
	Living situation: alone	,236	,740	,449	1,219
Very good	Constant	,001			
	Gender: male	,857	1,039	,684	1,578
	Nationality (ref. Norwegian)				
	German	,217	,263	,032	2,196
	Hungarian	,014	,081	,011	,601
	Financial situation (ref. often/daily problems)				
	No/hardly any financial problems	,000	2,788	1,590	4,891
	Sometimes problems	,007	2,538	1,296	4,973
	Study place (ref. Budapest)				
	Dresden	,768	,868	,338	2,226
	Munich	,952	,969	,349	2,686
	Pécs	,419	1,230	,745	2,030
	Living situation: alone	,081	,647	,397	1,054
Excellent	Constant	,015			
	Gender: male	,127	1,402	,909	2,164
	Nationality (ref. Norwegian)				
	German	,166	,222	,027	1,864
	Hungarian	,004	,052	,007	,390
	Financial situation (ref. often/daily problems)				
	No/hardly any financial problems	,000	4,092	2,168	7,724
	Sometimes problems	,016	2,515	1,184	5,344
	Study place (ref. Budapest)				
	Dresden	,242	,562	,214	1,475
	Munich	,884	,926	,328	2,610
	Pécs	,132	1,494	,886	2,521
	Living situation: alone	,503	,842	,509	1,393

a. reference category: poor/fair health

### Health promotion and associated factors

Significantly differentiated response behaviour regarding health promotion belief was detected, measured by a question on how much students consider currently being able to do (“can do”) for their health (chi^2^-test, *p* ≤ 0.001): The vast majority of Hungarian students (95.3 %) vs. 67.4 % of German and 56.7 % of Norwegian students reported that they currently “can do” *very much*/*a lot* for their health. In contrast, considerably more Norwegian (73.0 %) than Hungarian (63.7 %) or German students (51.5 %) reported that they currently do (“really do”) *very much*/*a lot* for their health (chi^2^-test, *p* ≤ 0.001, see Table 3).

**Table 3:** Health promotion belief and activities of medical students with different nationalities

	How much can you do or really do for your health currently?							
	Very much		A lot		Not much		Nothing at all	
	Can do	Really do	Can do	Really do	Can do	Really do	Can do	Really do
German students (*N* = 1289)	14.4 %	5.1 %	53.0 %	46.4 %	30.6 %	45.6 %	1.9 %	2.8 %
Hungarian students (*N* = 1057)	42.7 %	9.5 %	52.6 %	54.2 %	4.7 %	35.5 %	0.0 %	0.8 %
Norwegian students (*N* = 148)	13.5 %	8.1 %	43.2 %	66.9 %	41.9 %	22.3 %	1.4 %	2.7 %

#### Socio-demographic factors

There were no significant correlations between age and assessment of health promotion belief (“can do”, r_spearman_ = 0.01/*p* ≥ 0.05) or current health promotion activities (“really do”, r_spearman_ = 0.003/*p* ≥ 0.05). Depending on gender, similar answer distributions regarding health promotion belief (“can do” *very much*/*a lot*: m: 79.8 % vs. f: 78.0 %) and current health promotion activities (“really do” *very much*/*a lot*, m: 57.0 % vs. f: 58.8 %) were observed. These small gender-related differences were significant due to the sample size (chi^2^-test, *p* ≤ 0.05).

#### Study context

We found that significantly more students at Hungarian study sites (Pécs 85.5 %/Budapest 84.6 %) compared to German study sites (Dresden 70.1 %/Munich 66.0 %) reported that they currently “can do” *very much*/*a lot* for their health (chi^2^-test, *p* ≤ 0.001). Regarding current health promotion activities, more students at Hungarian study sites (Pécs 65.4 %/Budapest 61.0 %) compared to German study sites (Dresden 51.6 %/Munich 48.9 %) assessed to “do currently” *very much*/*a lot* for their health (chi^2^-test, *p* ≤ 0.001). Significantly more German students in Budapest (51.2 %) compared to other study sites (25.6-34.0 %) thought they “can do” *not much/nothing at all* for their health (chi^2^-test, *p* ≤ 0.001). The study site affected the current health promotion activities (“really do”) only in the German student sample (chi^2^-test, *p* ≤ 0.01): significantly less German students in Pécs (38.4 %) reported to currently do *not much/nothing* for their health compared to Germans at the other study sites (48.4–55.9 %).

Significant but weak correlations between assessment of health promotion belief (“can do”) and study year (r_spearman_ = −0.135/*p* ≤ 0.001) were detected: A higher number of students in advanced study years (84.7 % of 3rd and 82.4 % of 5th vs. 72.8 % of 1st) reported that they currently “can do” *a lo*t/*very much* for their health. The correlation between study year and current health promotion activities (“really do”) was also significant but weak (r_spearman_ = −0.057/*p* ≤ 0.001). The distribution of answers was similar; the proportions of students claiming to “do currently” *a lot*/ *very much* for their health in advanced study years were slightly higher (64.4 % of 3rd and 57.8 % of 5th vs. 53.4 % of 1st study year).

#### Socio-cultural factors

A significant difference in health promotion belief (“can do”) among students was detected in relation to the living situation (chi^2^-test, *p* ≤ 0.001): 80.9 % of students living together with at least one person reported that they currently “can do” *a lot*/*very much* for their health. This is a considerably higher number than that of students living alone (72.6 %). Contrarily, no significant association between living situation and current health promotion activities was observed (chi^2^-test, *p* ≥ 0.05). Furthermore, no significant associations were found between religiousness and health promotion belief (“can do”) or current health promotion activities (“really do”) (chi^2^-test, *p* ≥ 0.05). We detected significant associations between financial situation and current health promotion activities (chi^2^-test, *p* ≤ 0.001) but none regarding health promotion belief (chi^2^-test, *p* ≥ 0.05). Medical students describing *often/daily* problematic financial situations reported in significantly smaller numbers that they currently do *a lot*/*very much* for their health (50.5 % vs. 60.2 % with *no*/ *hardly any* problems).

#### Predictors of health promotion in multivariable models

Multinominal logistic regression models regarding 1) health promotion belief (“can do”) and 2) current health promotion activities (“really do”) were conducted including each significantly associated variables of the bivariate analysis (see above).

Similar predictors were detected for students’ assessment “can do” *very much*/*a lot* compared to “can do” *not much/nothing* for their health currently (Table 4). A Hungarian (OR ≥ 14.9) and German (OR ≥ 1.7) nationality significantly increased the odds, as did studying in Pécs (OR ≥ 1.7). The first study year was significantly associated with lower odds to report “can currently do” *very much* (OR = 0.4) or *a lot* (OR = 0.6) health-wise. The male gender increased the health promotion belief in the model significantly but only slightly (OR = 1.4), and only for the answer option “can do” *very much* compared to “can do” *not much/nothing*. The extent of the model forecast with included sociocultural factors was 54.2 %, the highest forecast concerned the answer option *a lot* with 87.1 %. The significant predictors could explain 23.8 % of the total variance of health promotion belief.

**Table 4:** Predictors of health promotion belief based on a multinominal regression model

How much can you do for your health currently? ^a^		Significance	Exp(B)	95 % confidence interval Exp(B)	
				Lower limit	Upper limit
Very much (*n* = 623)	Constant	,000			
	Gender: male	,033	1,350	1,024	1,779
	Nationality (ref. Norwegian)				
	German	,037	2,027	1,043	3,940
	Hungarian	,000	49,366	25,846	94,287
	Study year (ref. 5th study year)				
	1st study vear	,000	,414	,291	,588
	3rd study year	,054	1,454	,994	2,126
	Study place (ref. Budapest)				
	Dresden	,298	1,316	,784	2,209
	Munich	,383	1,270	,742	2,174
	Pécs	,001	1,934	1,313	2,848
	Living situation: alone	,334	1,161	,857	1,573
A lot (*n* = 1255)	Constant	,270			
	Gender: male	,210	1,160	,920	1,463
	Nationality (ref. Norwegian)				
	German	,030	1,673	1,050	2,665
	Hungarian	,000	14,951	9,081	24,615
	Study year (ref. 5th study year)				
	1st study vear	,001	,617	,458	,831
	3rd study year	,106	1,317	,943	1,839
	Study place (ref. Budapest)				
	Dresden	,017	1,601	1,089	2,353
	Munich	,194	1,302	,875	1,937
	Pécs	,002	1,739	1,225	2,470
	Living situation: alone	,786	1,034	,813	1,315

a. reference category: not much or nothing at all

All of the included independent variables in the multinominal logistic regression model regarding the current health promotion activities were significant for the total model (chi^2^/*p* ≤ 0.001). Nevertheless, the fit of the model was very weak (Nagelkerkes R^2^ = 0.055). The extent of the model forecast with included sociocultural factors was 55.7 %; the highest forecast concerned the answer option “can do” *a lot* with 76.4 %. Regarding the students’ assessment to do currently *very much* for their own health compared to *not much/nothing*, the chance was significantly increased in medical students studying at Pécs (OR = 1.6). The chance for an assessment to currently do *a lot* health-wise compared to *not much/nothing* was significantly increased in students of the 3rd study year (OR = 1.3) and in students with *no*/*hardly any* financial problems (OR = 1.6). In contrast, male gender (OR = 0.8), German (OR = 0.5) or Hungarian nationality (OR = 0.7) did significantly decrease the chance for an assessment to “really do” *a lot* for one’s health compared to *not much/nothing* (Table 5).

**Table 5:** Predictors of health promotion activities based on a multinominal regression model

How much do you currently do for your health? ^a^		Significance	Exp(B)	95 % confidence interval Exp(B)	
				Lower limit	Upper limit
Very much	Constant	,000			
	Gender: male	,075	1,363	,969	1,917
	Nationality (ref. Norwegian)				
	German	,323	,649	,276	1,530
	Hungarian	,862	1,069	,503	2,272
	Study year (ref. 5th study year)				
	1st study vear	,249	,772	,497	1,199
	3rd study year	,261	1,298	,824	2,044
	Study place (ref. Budapest)				
	Dresden	,407	,751	,382	1,477
	Munich	,144	,571	,270	1,210
	Pécs	,029	1,573	1,048	2,359
	Financial situation (ref. often/daily problems)				
	No/hardly any financial problems	,105	1,774	,888	3,545
	Sometimes problems	,763	1,127	,518	2,455
A lot	Constant	,109			
	Gender: male	,037	,826	,690	,989
	Nationality (ref. Norwegian)				
	German	,002	,468	,292	,751
	Hungarian	,048	,653	,428	,997
	Study year (ref. 5th study year)				
	1st study vear	,273	,882	,705	1,104
	3rd study year	,027	1,314	1,032	1,673
	Study place (ref. Budapest)				
	Dresden	,783	,954	,684	1,332
	Munich	,321	,838	,591	1,188
	Pécs	,097	1,218	,965	1,536
	Financial situation (ref. often/daily problems)				
	No/hardly any financial problems	,005	1,601	1,156	2,217
	Sometimes problems	,286	1,217	,848	1,746

a. reference category: not much or nothing at all

## Discussion

Our study showed that the majority of the medical students assessed their health status as very good or excellent, as expected, based on the age group and an assumably high level of health knowledge. Nevertheless, differences regarding nationalities were observed: The analysis of the subpopulations (German, Hungarian and Norwegian medical students) showed that health status was mostly affected by financial situation and by being of Hungarian nationality. Association of health status and financial situation is already well known [26]. Our study showed that Hungarian students assessed their health status worse than students from Germany (studying either in Germany or Hungary) or Norway (studying in Hungary). Previous studies among medical students from Hungary and the Czech Republic have shown similar effects compared to Swiss, German and British students [14, 15]. One explanation could be a “healthy student” effect among those who decide to study abroad. But the results of our study based on the sample of Germans studying in either Germany or Hungary did not confirm this: there were no significant differences regarding health status depending on study site.

Regarding health promotion belief there were great nationality-based differences. Nearly all (95.3 %) of the Hungarian students reported they can currently do *a lot/very much* for their health, the proportion was by far higher than that of Germans (67.4 %) and Norwegians (56.7 %). Also, an advanced stage of studies and the study site Pécs were positively associated with health promotion belief. However, the effect of studying in one’s home country versus abroad could only be analyzed among Germans: A significant number of German students in Budapest thought they can do *not much/nothing at all* (51.2 %) for their health, compared to Germans at other study sites (25.6–34.0 %).

Based on our study, the belief in health promotion did not correlate with current health-promoting behaviors: although more Hungarian students believed they can do a *lot/very much* for their health, more Norwegian students reported actually promoting their health a *lot/very much*. The study site had a stronger effect on positive health promotion belief but also on current activities: Students in Pécs reported more often being currently able to do - and also doing – a *lot/very much* for their health. This was also confirmed in the subgroup of German students but there were differences between the Hungarian study sites: the proportion of students reporting doing *not much/nothing* was lowest for German students in Pécs (38.4 %) and highest in Budapest (55.9 %).

One explanation for the positive effects of studying in Pécs could be the bigger amount of obligatory physical education in the curriculum: 2 h of sport courses a week are obligatory for all medical students during 4 out of the first 10 semesters, and these are offered throughout the studies with the aim to promote regular fitness and a healthy way of life [27]. To compare, obligatory courses are also offered in Budapest but only for 1 h/week [28]. At German study sites sport courses are voluntary. There is evidence that health promotion at a study site can be very effective: Even a one semester attendance in an elective course on relaxation techniques reduced burnout and anxiety among medical students significantly [29].

### Limitations

Although the total response rate was satisfactory, there was regional variation. Due to the study design we could not explain all the results, e.g. the reason why Hungarian students rate their own health lower than other students, and also the reasons why Hungarian students believe they can do a lot for their health but report doing not that much for it. The impact of further factors that were excluded in our analysis, such as the presence of chronic diseases, mental health and risk behaviour, should be analyzed in further studies. Recall bias and response bias could not be ruled out because of self-reported data. We aimed to minimize social desirability, a common bias regarding self-reported abilities or sensible topics, by securing the anonymity of participants.

## Conclusions

Based on our study, gender and study year played only a minor role for health status and health promotion beliefs and activities of medical students. Structural (study site) and somewhat socio-cultural factors (nationality, financial situation) explained in particular the differences in health promoting behaviors (s. psychosocial model). We argue that situational prevention and corporate social responsibility should be highlighted further to support health promoting behaviors in study settings. The first step could be to include obligatory, free-of-charge courses for health promotion (activity and relaxation) in study curriculum for all students, following the Hungarian model. In case of medical students, this could help students to cope with the high requirements of medical studies and foster long-lasting health effects affecting the health of (future) physicians and indirectly their patients.
